# How channel elasticity enhances and directs flow in dendritic microfluidic networks

**DOI:** 10.1038/s41378-025-01078-z

**Published:** 2025-12-24

**Authors:** Efstathios Mitropoulos, Claas-Hendrik Stamp, Arwin Marbini, Sebastian Milster, Joachim Dzubiella, Thomas Pfohl

**Affiliations:** 1 Physikalisches Institut, Albert-Ludwigs-Universität Freiburg, Freiburg, Germany; 2 Cluster of Excellence livMatS, Albert-Ludwigs-Universität Freiburg, FIT – Freiburg Center for Interactive Materials and Bioinspired Technologies, Freiburg, Germany

**Keywords:** Physics, Engineering, Materials science

## Abstract

Pressure drop per unit length is key to limiting the magnitude of flow in vascular systems and fluidic devices. This study presents a straightforward, pressure-responsive method to enhance flow compliance in dendritic microfluidic systems by manipulating the local elasticity. A series of dendritic fluidic networks with varying numbers of elastic elements were developed using replica molding of the elastomer polydimethylsiloxane in a single fabrication step. These elements, consisting of thin elastic membranes, deform under pressure, unlike rigid walls. The geometry and hydrodynamic properties of the networks were characterized by flow velocity measurements and fluorescence microscopy. The most elastic network showed a significant increase in compliance with thin membranes replacing rigid walls, resulting in a non-linear increase in flow rate. Selective placement of elastic elements allowed pressure-controlled flow directionality. This approach reduces pressure loss, does not require complicated fabrication steps, and allows dynamic flow manipulation in specific regions of microfluidic networks.

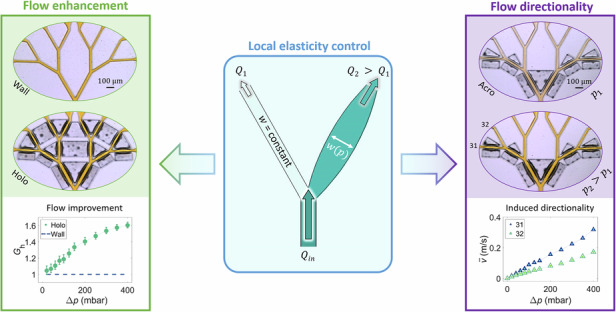

## Introduction

Ever since its introduction, the field of microfluidics has been used both as a tool to study different scientific phenomena and as a platform to bring practical applications to the public. From in vitro analogs of the human body^1,2^, drug development and delivery^3–6^, promising disease detection devices^7,8^, cell cultures, bacterial colonies, biofilms^9–13^ and polymerase chain reaction (PCR) chips^14,15^, to devices able to trap and isolate nano-particles^16,17^ or devices used to study the effects of good solvents on polymers^18^ and even an impressive array of electronic-like fluidic elements^19–22^, microfluidics has found use in an astonishing number of disciplines. Their widespread use can be attributed to the benefits they offer to researchers and the industry alike. Microfluidic devices are advantageous because of their small size and increased surface-to-volume ratio, allowing for increased chemical reaction speeds and efficiency. Their size and advances in microfabrication technologies allow for cost-effective integration of multiple functions on a single chip, while parallelization and high throughput can be achieved during assembly^20,21,23,24^.

Despite its many advantages, microfluidics has one significant drawback. Unlike electronics, with which it shares a number of similarities and merits^25,26^, the fundamental physics of microfluidics indicates that it suffers from a significant potential drop along the length of its channels^23,27^, especially as channel dimensions decrease and devices are connected in series. This disadvantage can cause the typically low-power microfluidic devices to require a considerable amount of energy to operate. Usually, a trade-off between required energy and functionality can be achieved. However, there is a general desire to reduce the dimensions as much as possible, both to increase the ever-important surface-to-volume ratio and to safely avoid non-laminar flow conditions.

One of the parameters usually affecting the performance and behavior of microfluidic devices is the hydrodynamic resistance^18,28–35^. Typical ways of controlling the hydrodynamic resistance include the geometry of channels^20,21,23^, properties of working fluids^23,24,27^, multiple (and sometimes functionalized) layers^16,17,33–36^ and combinations of these methods^18,28,32^. Among these methods, the introduction of thin membranes as additional layers in the devices is one of the earliest and most widespread. Membranes in between other device layers have been used to replicate physiological behavior in organ-on-chip applications^1,36^, to create effective microfluidic valves and switches^33,37–39^ and even to design microfluidic analogs of complex electronic elements^19–21^ and microfluidic pumps^40–42^. Despite the admittedly impressive range of applications, the idea of membranes sandwiched between layers has some disadvantages. Firstly, the fabrication of individual membrane layers can itself be a complicated and time-consuming process. In addition, the deflection of these membranes usually requires additional external stimuli sources of actuation that do not contribute to the functionality of the device in any other way, such as additional pressure sources, hydrogels, fluids, heat sources or even circuitry^36,38,39,43^. Finally, sandwiching membranes between other layers increases the complexity associated with the assembly and alignment of the device.

In this work, we present a straightforward method that eliminates the drawbacks associated with the introduction of membranes as additional device layers, using standard replica molding techniques to reduce the hydrodynamic resistance of microfluidic devices in a pressure-responsive manner. Thin, high aspect ratio membranes replacing channel walls adjust the local elasticity of the microfluidic channels. A more complex dendritic channel network is used to demonstrate the applicability of the method in scenarios beyond the confines of single, straight channel designs. Particle image velocimetry (PIV) experiments revealed an overall increase in flow rate due to the incorporation of more elastic designs. The deformation of the membranes during pressure-driven flow is measured using fluorescence microscopy to correlate this with an overall increase in flow rate. The implementation of thin membranes on specific channel branches of a modified dendritic channel network, results in flow directionality in this network. Reducing the hydrodynamic resistance by controlling the local elasticity decreases the pumping power required by microfluidic systems. The pressure-responsive flow directionality can lead to precise, non-symmetric flow rates in different regions of microfluidic devices.

## Results and discussions

### Implementation of local elasticity

Flow through a channel follows a flow-impedance relationship. This relationship, linking flow rate $Q$ and applied pressure drop ${{\Delta}{p}}$ between the inlet and outlet of the channel^23,24,27^, is given as

1 $$Q=\frac{\Delta p}{{R}_{{\rm{h}}}}$$

where ${R}_{{\rm{h}}}$ is the hydrodynamic resistance of the system, a parameter indicating the impedance of the system to flow through it. ${R}_{{\rm{h}}}$ depends on the geometry of the channel system and the properties of the working fluid. For microfluidic channels with a rectangular cross-section, where the width $w$ is significantly smaller than the height $h$, ${R}_{{\rm{h}}}$ can be approximated as^23^

2 $${R}_{\rm{h}}\mbox{}=\mbox{}\frac{12\eta L}{h{w}^{3}(1-0.63\frac{w}{h})}$$

where $\eta$ is the viscosity of the fluid and $L$ the length of the channel. It becomes apparent from Eq. 2 that an increase in $w$ will result in a decrease in ${R}_{{\rm{h}}}$, which in turn, based on Eq. 1, will result in higher $Q$ values. If this increase in $w$ follows changes in the applied pressure $\Delta p$, then a pressure-responsive mechanism can be introduced to increase the flow rate *Q* non-linearly.

Introducing membranes or thin plates as local flow boundaries, instead of rigid walls, is an attractive method of controlling local elasticity. This method is well-described by standard plate theory^44^. For a thin plate whose thickness *c* is significantly smaller than its length $a$ and height $b$, the maximum deformation ${d}_{\max }$ at the center of the plate under hydrostatic pressure conditions (a pressure drop that increases linearly along the length of the plate) for $a\gg b$ is given as

3 $${d}_{\max }=\alpha \frac{{p}_{\max }{b}^{4}}{D}$$

where ${p}_{\max }$ is the maximum pressure value applied on the plate, $\alpha$ is a numerical parameter incorporating the geometrical and edge properties of the plate and $D$ is its structural rigidity defined as

4 $$D=\frac{E{c}^{3}}{12\left(1-{\nu }^{2}\right)}$$

where $E$ is the Young’s modulus of the plate material and $\nu$ is Poisson’s ratio. The terms plate and membrane are used interchangeably herein.

A channel of width $w$ defined by fixed non-moveable boundaries (such as rigid walls) is assumed in Eq. 2. If some of these rigid non-deformable walls are replaced by thin deformable membranes, then an increase of the maximum pressure at the inlet of the channel ${p}_{\max }$ will directly lead to a deformation of membranes in accordance with Eq. 3. The deformation of the membranes would result in a higher $Q$ for the same $\Delta p$ (Eq. 1)^22,45,46^.

To introduce thin membranes instead of rigid walls as the boundaries of the microfluidic channels in a single fabrication step, cavities are used in the vicinity of the channels. By placing these cavities close enough to a channel, thin membranes can be formed between the channel and the cavities.

**Fig. 1:**
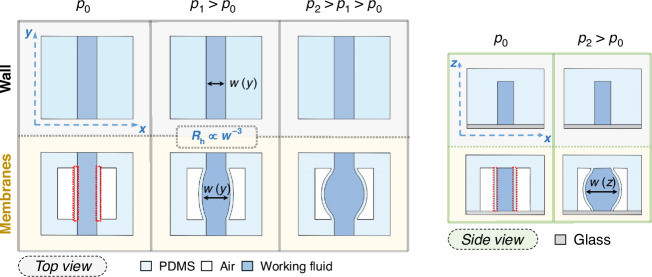
Control of local elasticity. Top view (left) and side view (right) of the design-based approach for controlling local elasticity. Devices with rigid walls do not show significant changes in channel geometry when pressure is applied to the working fluid. The incorporation of thin membranes (highlighted by red boxes) via air-filled cavities adjacent to the channels leads to an increase of the channel width $w$ as the working pressure increases, resulting in a decrease in the hydrodynamic resistance *R*_h_

These thin membranes can now influence the local elasticity of the channel, allowing for the desired pressure-responsive enhancement of the flow through it, which is shown in Fig. 1. For a pressure ${p}_{0}$ no significant change is observed in the case of rigid walls and membrane-walled channels. At an increased pressure ${p}_{1}$, the channel with rigid walls retains its shape, whereas the channel surrounded by membranes exhibits an overall increase in width. By applying an even higher pressure ${p}_{2}$, the width of the more elastic channel continues to increase, while the geometry of the less elastic channel is not affected.

Note that the deflection of the membrane is not homogeneous along its length and height. For all membranes in this work, $c=20\,{\upmu }\mbox{m}$ and $b=h=200\,{\upmu }\mbox{m}$. Polydimethylsiloxane, PDMS, with a monomer-linker weight ratio of 10:1 is the material used for the microfluidic devices and hence the membranes. The material has a reported Young’s modulus of $E\cong 1.7\,{\rm{MPa}}$ and a reported Poisson’s ratio of $\nu \cong 0.5$^47–51^. All devices were fabricated by replica molding (see Methods) and were covalently bonded to a glass slide for optical access.

### The design of dendritic wall and holoelastic fluid networks

**Fig. 2:**
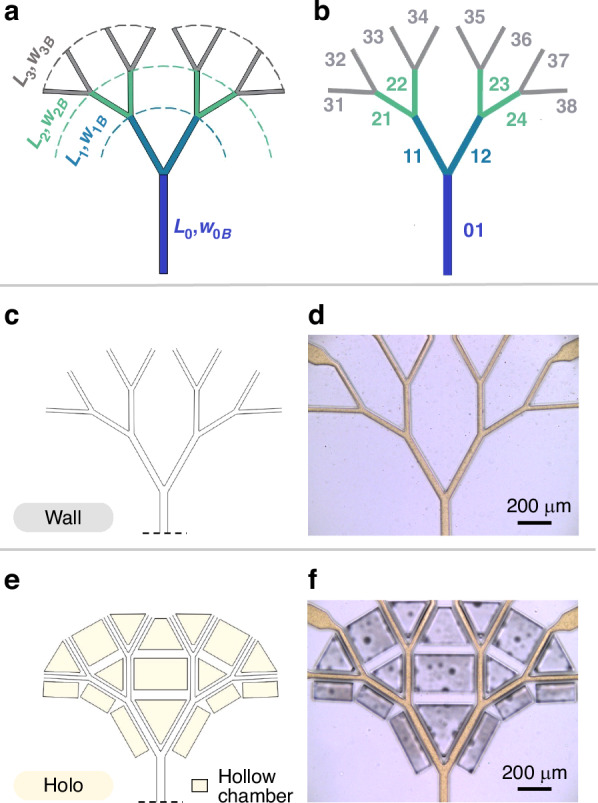
Designs of dendritic wall and holoelastic fluid networks. **a** Length *L* and width *w* for each level of the dendritic channel network. Width values are based on Murray’s rule of bifurcations^52–54^. **b** Indices of all branches of the dendritic network. **c** Design of a device with rigid walls and **d** the fabricated device after PDMS replica molding, called wall device. **e** Design of a device with membranes on all branches after level 0 and **f** the fabricated device after replica molding, called holoelastic (holo) device. The yellow color is due to the aqueous fluorescein sodium salt solution used as the working fluid. In **c**, **e** the dotted lines indicate that the full length of the channel is trimmed in the sketch

To characterize the effectiveness and applicability of the design-based approach for non-trivial, physiologically and naturally relevant systems^52–54^, a one to eight-point dendritic system consisting of bifurcating channels was chosen. A single channel branches into two channels, which branch into four channels, which in turn branch in eight channels. As such, each bifurcation gives rise to a new dendritic level. The first parent branch is also the first dendritic level, level 0. The width of each channel branch in the dendritic structure is denoted by ${w}_{S,B}$, where $S$ is the level of the dendritic structure and $B$ the number of the branch for that level, counting from left to right. Both $S$ and $B$ take on integer values with $0\le S\le 3$ and $1\le B\le {2}^{S}$. The width of each channel for the different levels is based on Murray’s rule of bifurcations^52–54^ with ${w}_{S+1}/{w}_{S}=\root{3}\of{2}$, where ${w}_{0}=50\,{\upmu }\mbox{m}$. The lengths referring to the channels of each level are denoted as ${L}_{S}$ according to the previous notation and are chosen as designer parameters (${L}_{0}=678\,{\rm{\mu m}}$, ${L}_{1}=366\,{\rm{\mu m}}$, ${L}_{2}=205\,{\rm{\mu m}}$, ${L}_{3}=228\,{\rm{\mu m}}$). The bifurcation angle $\theta =59^\circ$ is also chosen as a designer parameter and is within the range of observable bifurcation angles in organisms^52^. An overview of the lengths and widths of the different dendritic levels can be seen in Fig. 2a, while a full visualization of the branch indexing over the dendritic design is shown in Fig. 2b.

Hereafter, a dendritic device with its channels embedded in the bulk material without any thin elastic membranes will be referred to as a ‘’wall” device. A top view sketch of the channel system of the wall device is shown in Fig. 2c. A transmission microscopy image of the final bonded wall device after PDMS replica molding is shown in Fig. 2d. The working fluid in the channels of the device is an aqueous fluorescein sodium salt solution.

The design of devices with implemented local elasticity control for all their branches is one in which cavities are introduced around the sides of all the channels subsequent to the initial parent channel. Such devices are referred to throughout this work as “holoelastic” or “holo” (from the Greek word όλο, “holo”, meaning whole or entire) devices. A top view sketch of the channel system of the holoelastic device is shown in Fig. 2e, while a transmission microscopy image of the bonded holoelastic device filled with the working fluid is shown in Fig. 2f.

This way of introducing local elasticity eliminates the need for multiple functionalized layers and externally pressurized regions on the device and allows for the fabrication of the dendritic device in a single replica molding step (see Methods). Some aqueous droplets can be observed in the hollow chambers in Fig. 2f. Their presence is associated with perfusion flows from the channels through the membranes during the experiments^55^.

### Dendritic wall and holoelastic fluidic networks under different pressure conditions

**Fig. 3:**
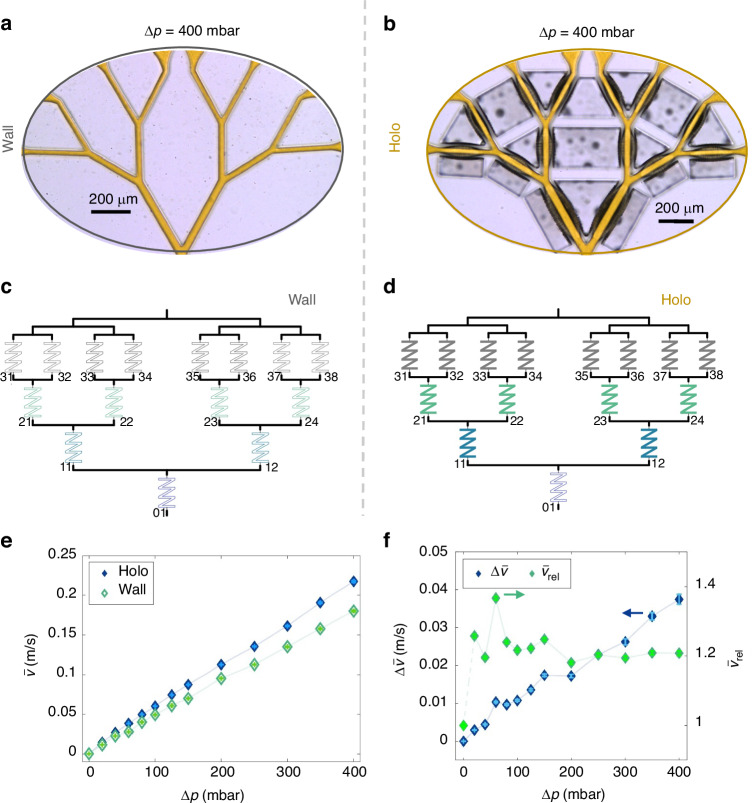
Comparison of wall and holoelastic devices. Transmission microscopy image showing the behavior of **a** the wall device and **b** the holoelastic device at $\Delta p=400\,\mbox{mbar}$. Electric analog for hydrodynamic resistances for **c** the wall device and for **d** the holoelastic device. Color-filled resistors indicate a pressure-responsive hydrodynamic resistance. Indices ${SB}$ indicate the level and branch to which the hydrodynamic resistance refers. **e** Averaged velocity at the outlets for the wall and holoelastic devices as measured by PIV. **f** Increase of the velocity difference $\Delta \bar{v}$ (left *y*-axis) and average of relative velocity ${\bar{v}}_{\rm{rel}}$ (right *y*-axis) between the wall and holoelastic devices. The first point for ${\bar{v}}_{\rm{rel}}$ (different color) is set to 1 by definition

Transmission microscopy images of wall and holoelastic devices under an applied pressure difference of $\Delta p=400\,{\rm{mbar}}$ are shown in Fig. 3a, b. The pressure differences were supplied by an OBI-I pressure pump (Elveflow, Paris, France). By comparing the channel geometry of the dendritic wall fluidic device at $\Delta p=0\,{\rm{mbar}}$ (see Fig. 2d) and $\Delta p=400\,{\rm{mbar}}$ (see Fig. 3a) virtually no changes can be observed. In contrast, for the holoelastic device, in the case of $\Delta p=400\,\mbox{mbar}$ (see Fig. 3b), the membranes surrounding the channels show a significant deformation, increasing the overall width of the channels when compared to the behavior at $\Delta p=0\,\mbox{mbar}$ (see Fig. 2f). The electrical analogs for the hydrodynamic resistance of the walls and holoelastic devices are shown in Fig. 3c, d. The resistance values in Fig. 3c are independent of pressure changes, whereas those in Fig. 3d are not.

Particle image velocimetry (PIV) measurements were conducted over a pressure range from 0 to 400 mbar to gain insight into how much the change in channel geometry can affect the overall flow behavior of the devices. For both the wall and holoelastic devices, PIV measurements were taken in wider areas at the end of the third level of the device. These areas were around twenty times wider than the level 3 branches. The volume flow rate is conserved between wider and narrower areas of channels connected in series and ${Q=v}\,\cdot\,{w}\,\cdot\,{h}$, where $v$ is the mean velocity of the fluid. A narrower channel leads to higher mean velocity values $v$ for the fluid. This setup was chosen because it was impossible to track tracer particles in the narrow channels of the dendritic design with the equipment used.

For each device, the measured velocities for the branches are averaged resulting in $\bar{v}$, which is plotted against $\Delta p$ (Fig. 3e). For small pressure differences, the average velocity values for both devices appear close, but as $\Delta p$ increases the average velocity of the holoelastic device is consistently higher than that of the wall device for the same pressure conditions. To study this behavior further, both the absolute difference between the average velocity values of the holoelastic and wall devices $\Delta \bar{v}={\bar{v}}_{\rm{holo}}-{\bar{v}}_{\rm{wall}}$ and the relative velocities between the two devices ${\bar{v}}_{\rm{rel}}={\bar{v}}_{\rm{holo}}/{\bar{v}}_{\rm{wall}}$ were calculated and plotted for each $\Delta p$ value. The resulting plot is shown in Fig. 3f. $\Delta \bar{v}$ presents an increasing trend over $\Delta p$ from 0 m/s up to 0.04 m/s, ${\bar{v}}_{\rm{rel}}$ over $\Delta p$ is in the range of 1.2–1.3. These results reveal that the change in geometry caused by the deformation of the membranes due to the applied pressure has two effects in the case of the holoelastic devices. First, it enhances the flow velocity compared to the wall device for similar $\Delta p$ values, even in areas further down the dendritic structure. In addition, this velocity enhancement increases with $\Delta p$, indicating a pressure-sensitive response mechanism. To better understand the mechanism responsible, fluorescence microscopy experiments were carried out by keeping the microscope focused on the center plane, to measure the width of the channels at that position. This method allowed direct analysis of the morphology of the channels along their length at different ${\Delta}p$ values. For this set of experiments, the same pressure profiles were used as for the PIV measurements.

**Fig. 4:**
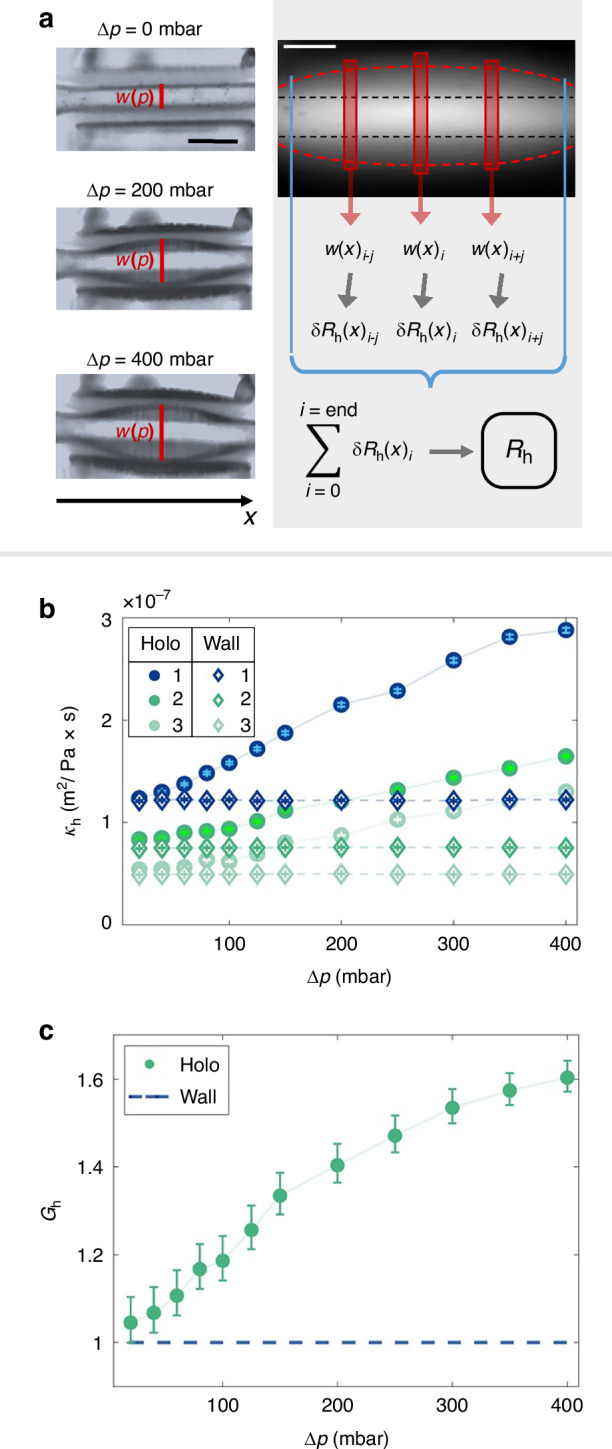
Flow magnitude comparisons between wall and holoelastic devices. **a** Left: Membrane deformation for the dendritic branch 31 at different ${\Delta}p$. Scale bar: 50 µm. Right: Schematic of the hydrodynamic resistance determination from a fluorescence image (black dashed lines: channel boundaries before deflection, red dashed lines: channel boundaries after deflection). Scale bar (white line): 50 µm. The local channel widths ${w(x)}_{i}$ were determined from the fluorescence intensity distribution and were converted to the corresponding hydrodynamic resistances $\updelta {R}_{{\rm{h}}}{(x)}_{i}$. Summing these values over the length of the entire branch gives its total resistance ${R}_{{\rm{h}}}$ (right). **b** Hydrodynamic conductivity ${\kappa }_{\rm{h},\it S}$ determined for the levels *S* = 1, 2 and 3 for the dendritic structures of the wall and holoeleastic devices based on fluorescence measurements of the channel width at the center of the device. Hydrodynamic conductivity is given in units of area over dynamic viscosity, indicating that an increase in ${\kappa }_{\rm{h}}$ means that a wider cross section is available for the fluid to flow through. **c** Improvement factor for the dendritic structure of the holoeleastic device compared to the wall device

The basic concept behind the fluorescence measurements is shown in Fig. 4a. On the left side of Fig. 4a, transmission microscopy images are shown. Optical effects due to membrane bending and droplet formation on the back of the membrane can be observed in the images for higher pressures. These optical effects make width measurements in transmission significantly harder and as such fluorescence measurements are used instead. A fluorescence image of a branch channel is shown on the right side of Fig. 4a. From the fluorescence intensity, the local width ${w(x)}_{i}$ of the channel was determined for small bands perpendicular to the channel direction as small as individual pixels $i$ over its entire length (channel morphology–red dashed line in Fig. 4a). Approximating these bands as narrow rectangular channels, Eq. 2 was used to convert these width values into corresponding hydrodynamic resistance values $\updelta {R}_{{\rm{h}}}{(x)}_{i}$. All contributions $\updelta {R}_{{\rm{h}}}{(x)}_{i}$ can be considered to be in series, therefore summing them over the entire length resulted in the total resistance ${R}_{{\rm{h}}}$ of the channel. In order to ascertain the effective width ${w}_{\rm{eff}}$, which is the width of a purely rectangular channel of same hydrodynamic resistance length and height as the channel under investigation, Eq. 2 was solved for $w\equiv {w}_{{\rm{eff}}}$ using the obtained ${R}_{{\rm{h}}}$. This approach neglects the bending of the membrane in the *z*-direction, yet remains adequate. Since the effective width ${w}_{\rm{eff}}$ is a value that can remain constant over the length of a channel, an analogy to electrical conductivity is introduced to describe the compliance of channels in a more general way. This analog is called hydrodynamic conductivity and is given as

5 $${\kappa }_{{\rm{h}}}=\frac{1}{{R}_{{\rm{h}}}}\frac{L}{A}$$

With constant cross-section $A={w}_{\rm{eff}}\,{\cdot}\,{h}$. In the case of the wall and holoelastic devices, the characteristic hydrodynamic conductivity values for each of the levels were obtained by averaging over all the different branches of the same dendritic level that measurements were conducted on.

The characteristic conductivities ${\kappa }_{\rm{h},\it S}$ of levels *S* = 1, 2 and 3 for the wall and holoelastic devices are plotted against ${\Delta}p$ in Fig. 4b. For low pressure values ${\kappa }_{\rm{h},1} > {\kappa }_{\rm{h},2} > {\kappa }_{\rm{h},3}$ is observed. This is primarily due to the decrease in channel width with increasing level. For low pressure values ($\Delta p=\mathrm{20,40}\,{\rm{mbar}}$), the conductivity values ${\kappa }_{\rm{h},\it S}$ for the same level $S$ for the wall and holoelastic devices are found to be almost identical. At $\Delta p=60\,\mbox{mbar}$, the holoelastic branches show an increased conductivity ${\kappa }_{\rm{h},\it S}$ compared to their wall counterparts. The hydrodynamic conductivity for the holoelastic branches further increases with increasing ${\Delta}p$. For $\Delta p > 200\,{\rm{mbar}}$, the conductivity of the holoelastic level 2 branches is higher than that of the of level 1 wall branches ${\kappa }_{\rm{h},2}^{\rm{holo}} > {\kappa }_{\rm{h},1}^{\rm{wall}}$, and similarly ${\kappa }_{\rm{h},3}^{\rm{holo}} > {\kappa }_{\rm{h},2}^{\rm{wall}}$. In the course of the experiment, only $A$ in Eq. 5 changes, while *h* remains constant. So, ${w}_{\rm{eff}}$ is the only factor that can affect the hydrodynamic conductivity. For the wall devices, ${\kappa }_{\rm{h},\it S}^{\rm{wall}}$ of each level $S$ remains largely constant for the studied ${\Delta}p$-range. In contrast, the deformation of the thin membranes in holoelastic devices, as indicated by Eq. 3, results in an increase of ${w}_{\rm{eff}}$ with increasing applied pressure ${\Delta}p$. This increase in ${w}_{\rm{eff}}$ is responsible for the pressure-dependent increase of ${\kappa }_{\rm{h},\it S}^{\rm{holo}}$ observed at all levels of the holoelastic device, as previous studies for differently designed single elastic channels would suggest^22,45,46^.

The hydrodynamic resistance ${R}_{{\rm{h}}}$ for each channel link in the dendritic structure was determined using fluorescence measurements. Using these ${R}_{{\rm{h}}}$ values, the total hydrodynamic resistance ${R}_{\rm{h},\rm{total}}$ for the entire dendritic structure was obtained for the wall and holoelastic devices for different $\Delta p$. This was achieved by employing the electrical resistance analogs for the wall and holoelastic devices, as shown in Fig. 3c, d in combination with Kirchhoff’s laws analog^23,25,26^.

It is evident that ${\kappa }_{\rm{h},\it S}^{\rm{wall}}$ exhibits minimal variability within the examined ${\Delta}p$-range for the wall devices (see Fig. 4b). Since the hydrodynamic resistance of each level branch remains constant and independent of ${\Delta}p$ at each level, ${R}_{\rm{h},\it SB}={R}_{\rm{h},\it S}$, we can estimate the total resistance of the dendritic wall network by

6 $${R}_{\rm{h},\rm{total}}^{\rm{wall}}=\mathop{\sum }\limits_{S=0}^{3}\frac{{R}_{{\rm{h}},S}}{{2}^{S}}$$

In comparison, the dendritic holoelastic networks show a pressure-dependent hydrodynamic resistance of each level branch, with ${R}_{{\rm{h}},{SB}}^{{\rm{M}}}={R}_{{\rm{h}},S}^{{\rm{M}}}$ for the levels $S=\mathrm{1,2,3}$ and constant hydrodynamic resistance ${R}_{{\rm{h}},0}$ for $S=0$. Therefore, we can estimate the total resistance of the dendritic holoelastic network by

7 $${R}_{\rm{h},\rm{total}}^{\rm{holo}}\left(\triangle p\right)=\frac{{R}_{{\rm{h}},0}}{{2}^{0}}+\mathop{\sum }\limits_{S=1}^{3}\frac{{R}_{{\rm{h}},S}^{{\rm{M}}}}{{2}^{S}}$$

In order to see how compliant the devices are to flow, the term of hydrodynamic conductance is introduced as ${K}_{\rm{h},\rm{total}}={\left({R}_{\rm{h},\rm{total}}\right)}^{-1}$. In addition, the total conductances for the wall and the holoelastic devices were normalized to the total conductance of the wall device and expressed as the improvement factor ${G}_{\rm{h}}^{\rm{holo}}={K}_{\rm{h},\rm{total}}^{\rm{holo}}/{K}_{\rm{h},\rm{total}}^{\rm{wall}}={R}_{\rm{h},\rm{total}}^{\rm{wall}}/{R}_{\rm{h},\rm{total}}^{\rm{holo}}$. ${G}_{\rm{h}}^{\rm{holo}}$ increases with pressure indicating that the holoelastic devices improve the flow more with increasing pressure compared to the wall devices (Fig. 4c). The maximum ${G}_{\rm{h}}^{\rm{holo}}$ value at $\Delta p=\,400\,{\rm{mbar}}$ is increased by about 60 % compared to the wall case. This is due to the fact that an increase in pressure results in an increase in $w$ or ${w}_{\rm{eff}}$ in all channels with thin membranes in the holoelastic device as one would expect for elastic deformable channels^22,45,46^.

### Directing the main flow: Dendritic acroelastic devices

**Fig. 5:**
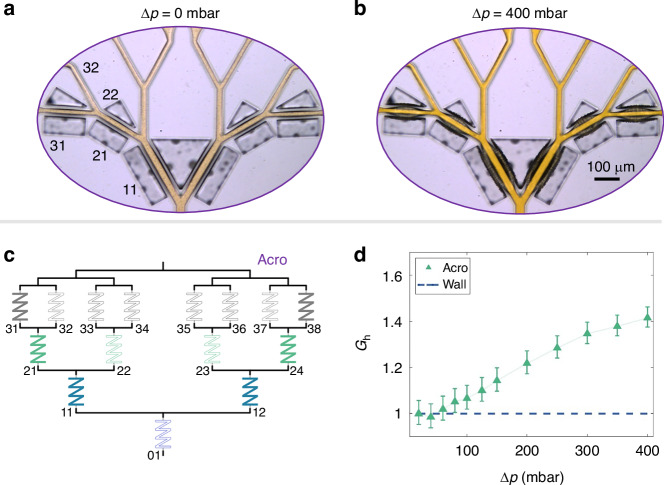
Design and properties of the dendritic acroelastic network. Transmission microscopy image showing the behavior of the acroelastic device at **a** $\Delta p=0\,\mbox{mbar}$ and **b** at $\Delta p=400\,\mbox{mbar}$. **c** Electric analog for hydrodynamic resistances for the acroelastic device. Color-filled resistors indicate a pressure-responsive hydrodynamic resistance. **d** Overall improvement factor for the dendritic structure in the acroelastic device

The different pressure-responsive behavior of wall and holoelastic devices is exploited in the design of a family of dendritic devices: These devices exhibit both increased flow compliance compared to the wall devices and pressure-induced preferential flow paths. A transmission microscopy image illustrating the behavior of such a device at ${\Delta}p=0\,\mbox{mbar}$ can be seen in Fig. 5a. In this particular device design, membranes are present exclusively in the outermost branches of the dendrite, with the rest of the channels being similar to those in the wall device. This device is referred to in this work as an “acroelastic” or “acro” (from the Greek word άκρο, “acro”, meaning side). A transmission microscopy image of the acroelastic device at ${\Delta}p=400\,\mbox{mbar}$ is shown in Fig. 5b. Significant deformations of the membranes can be observed in the elastic branches. These deformations result in a different channel geometry between branches at the same level, depending on whether they are surrounded by membranes or by rigid walls (e.g., branches 31, 32).

The analog electrical circuit for the hydrodynamic resistance of the acroelastic devices is shown in Fig. 5c. Hydrodynamic resistances ${R}_{{\rm{h}},31}^{{\rm{M}}}={R}_{{\rm{h}},38}^{{\rm{M}}}={R}_{{\rm{h}},3}^{{\rm{M}}}$, ${R}_{{\rm{h}},21}^{{\rm{M}}}={R}_{{\rm{h}},24}^{{\rm{M}}}={R}_{{\rm{h}},2}^{{\rm{M}}}$, ${R}_{{\rm{h}},11}^{{\rm{M}}}={R}_{{\rm{h}},12}^{{\rm{M}}}={R}_{{\rm{h}},1}^{{\rm{M}}}$ are pressure-dependent and the hydrodynamic resistances ${R}_{\rm{h},32}=\ldots ={R}_{\rm{h},37}={R}_{\rm{h},3}$, ${R}_{\rm{h},22}={R}_{\rm{h},23}={R}_{\rm{h},2}$, ${R}_{\rm{h},01}={R}_{\rm{h},0}$, are constant. We can estimate the total resistance of the dendritic acroelastic network by

8 $$\begin{array}{l}{R}_{\rm{h},\rm{total}}^{\rm{acro}}\left(\Delta p\right)=\frac{{R}_{{\rm{h}},0}}{{2}^{0}}+\frac{{R}_{{\rm{h}},1}^{{\rm{M}}}}{{2}^{1}}+{\frac{1}{2}\left[{\left(\frac{{R}_{{\rm{h}},3}^{{\rm{M}}}{R}_{{\rm{h}},3}}{{R}_{{\rm{h}},3}^{{\rm{M}}}+{R}_{{\rm{h}},3}}+{R}_{{\rm{h}},2}^{{\rm{M}}}\right)}^{-1}\right.}\\\qquad\qquad\qquad\qquad{\left.\,+\,{\left(\frac{{R}_{{\rm{h}},3}}{2}+{R}_{{\rm{h}},2}\right)}^{-1}\right]}^{-1}\end{array}$$

However, analysis of the fluorescence experiments allows for the experimental determination of the local hydrodynamic resistances of the dendritic acroelastic network and the calculation of the overall improvement factor ${G}_{\rm{h}}^{\rm{acro}}={K}_{\rm{h},\rm{total}}^{\rm{acro}}/{K}_{\rm{h},\rm{total}}^{\rm{wall}}={R}_{\rm{h},\rm{total}}^{\rm{wall}}/{R}_{\rm{h},\rm{total}}^{\rm{acro}}$ for different ${\Delta}p$ (Fig. 5d). For $\Delta p\lesssim 60\,\mbox{mbar}$, almost no change can be observed for the acroelastic device. As $\Delta p$ continues to increase, ${G}_{\rm{h}}^{\rm{acro}}$ also increases, reaching a value about 40 % higher than that of the wall device for $\Delta p=400\,\mbox{mbar}$. Nevertheless, the maximum value for the improvement factor (40 %) remains lower than the one for the holoelastic device (60 %) shown in Fig. 4c. The larger number of elastic elements in the holoelastic device is the reason for the different maximum improvement factor values between acroelastic and holoelastic devices.

**Fig. 6:**
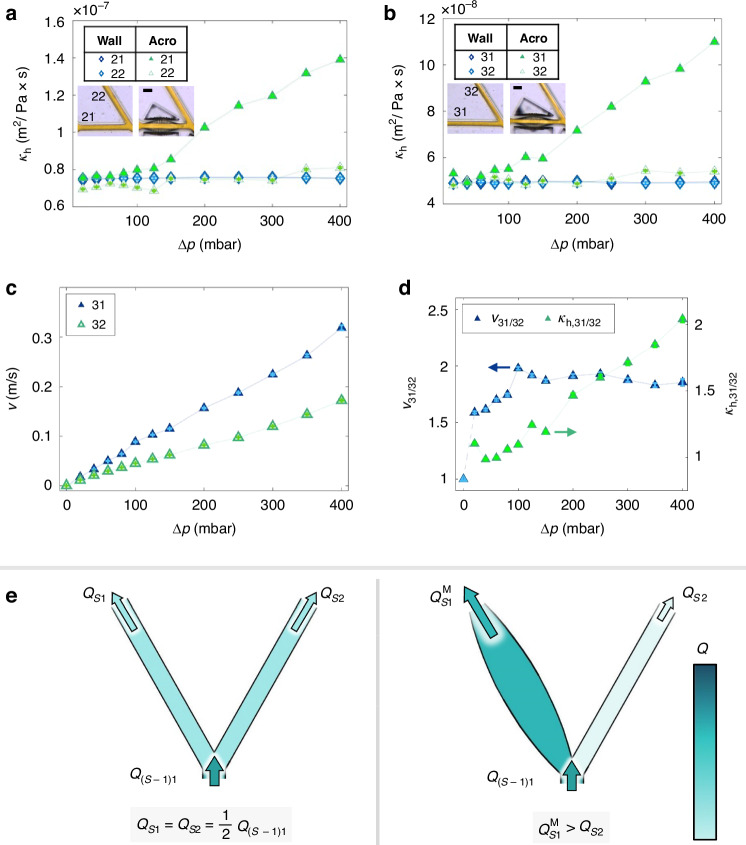
Flow directionality inducing mechanism in dendritic acroelastic devices. Hydrodynamic conductivity versus pressure for **a** branches 21 and 22 and **b** for branches 31 and 32 in the case of wall and acroelastic devices (Scale bar: 50 µm). Hydrodynamic conductivity is given in units of area over dynamic viscosity, indicating that an increase in ${\kappa }_{\rm{h}}$ means that a wider cross section is available for the fluid to flow through. **c** PIV velocity measurements versus pressure in the branches 31 and 32. **d** Relative velocities ${v}_{31/32}$ of branch 31 over 32 and relative hydrodynamic conductivity ${\kappa }_{{\rm{h}},31/32}$ between the same channels for the acroelastic device (arrows indicating respective *y*-axis for each quantity). The first point for ${v}_{31/32}$ (different color) is set to 1 by definition. **e** Sketch of the mechanism causing flow selection for a bifurcation

To better understand how this pressure-responsive increase in flow through the system was distributed throughout the dendritic acroelastic network, the determined conductivity values ${\kappa }_{{\rm{h}}}$ are plotted versus ${\Delta}p$ for the branch pairs 21, 22 and 31, 32 for the wall and acroelastic devices (Fig. 6a, b). These pairs are chosen because they are at the same dendritic level and have branches of varying elasticities. For $\Delta p\lesssim 60\,\mbox{mbar}$, the ${\kappa }_{{\rm{h}}}$ values for all branches at the same level are closely similar, for $\Delta p > 60\,\mbox{mbar}$ the more elastic branch of the each pair starts to show higher ${\kappa }_{{\rm{h}}}$ values than the other channel. With increasing pressure, the ${\kappa }_{{\rm{h}}}$ values of the more elastic branches continue to increase linearly, ${\kappa }_{{\rm{h}}}\propto \Delta p$. For these channels, the general relationship between the flow rate through them and the applied pressure difference across the branch is ${Q}_{{SB}}^{{\rm{M}}}\propto \Delta p(1+\beta \Delta p)$ with a channel dependent constant $\beta$ is observed (see Eqs. 1 and 5). Subsequently, under the condition that $1/\Delta p\ll \beta$, it can be deduced that ${Q}_{{SB}}^{{\rm{M}}}\propto {\Delta p}^{2}$. The ${\kappa }_{{\rm{h}}}$ values of the less elastic rigid-walled branches exhibit minimal variations with pressure. These observations indicate that for bifurcations in wall devices each parent channel always divides its flow rates almost equally between the bifurcation channels. For the acroelastic devices, an increased pressure in bifurcations with one more elastic and one less elastic element directs a greater fraction of the parent channel flow rate to the more elastic channel due to its higher ${\kappa }_{{\rm{h}}}$ value, e.g., about 1.75 at junction 21, 22 and 2.2 at junction 31, 32 at $\Delta p=400\,\mbox{mbar}$. Because of this effect, placing elastic elements along specific pathways in the dendritic structure can allow pressure-controlled flow preferentially along these paths.

PIV measurements are also conducted at the outlets of branches 31 and 32 of the acroelastic device. The measured velocities for each branch are plotted versus $\Delta p$ in Fig. 6c. As the pressure values increase, the flow velocities $v$ of branch 31 are higher than the velocity values of branch 32 for the same ${\Delta}p$. The higher $v$ values for branch 31 compared to branch 32 indicate that the increase in ${\kappa }_{{\rm{h}}}$ in the elastic parts of the dendrite can have a significant impact on the flow behavior of parts of the device that are further down the 8 end points of the dendrite itself. To track the increase in ${\kappa }_{{\rm{h}}}$ and $v$ between branches 31 and 32 the relative values of these values between these branches ${v}_{31/32}={v}_{31}/{v}_{32}$ and ${\kappa }_{{\rm{h}},31/32}={\kappa }_{{\rm{h}},31}/{\kappa }_{{\rm{h}},32}$ were calculated and plotted against ${\Delta}p$ (Fig. 6d). The pressure behavior of ${v}_{31/32}$ can be separated in two regions. For $\Delta p\lesssim 20\,{\rm{mbar}}\lesssim 80\,{\rm{mbar}}$, it shows a slight increase of up to around 1.7. For $\Delta p\gtrsim 100\,\mbox{mbar}$, its value stabilizes around 1.9. In the case of ${\kappa }_{{\rm{h}},31/32}$, its values increase with increasing pressure, indicating a pressure-responsive behavior of the dendritic structure. Note that ${v}_{31/32}$ and ${\kappa }_{{\rm{h}},31/32}$ do not exhibit the same values over different $\Delta p$, because unlike the ${\kappa }_{{\rm{h}},31/32}$ values, which are purely geometrical in nature, the ${v}_{31/32}$ values are also influenced by the hydrodynamic resistance of the inlet and outlet channels before and after the dendritic structure, due to flow conservation.

The maximum value for ${v}_{31/32}$ in the case of acroelastic branches (see Fig. 6d) of the same level is higher than ${\bar{v}}_{\rm{rel}}$ of holoelastic channels compared to wall channels (see Fig. 3f). The overall improvement factor of the acroelastic device is lower than that of the holoelastic device. Therefore, it can be inferred that a larger fraction of the total flow rate in the acroelastic case is directed via the more elastic pathways in the device.

A simplified concept of how pressure-responsive differences in flow conditions between channels at the same dendritic level can be used to induce flow preferentiality is illustrated in a sketch in Fig. 6e. It compares simple bifurcations of wall devices and acroelastic ones. In the case of the wall devices, flow conservation dictates that the amount of flow passing through the parent channel is divided equally ${Q}_{S1}={{Q}_{S2}=\frac{1}{2}Q}_{\left(S-1\right)1}$ between the bifurcating channels, which are identical in geometry. In the case of branching in the acroelastic device, for the same flow rate, a larger proportion will be directed to the pressure-responsive channel surrounded by membranes and a smaller proportion to the rigid-walled channel that maintains its dimensions (${Q}_{S1}^{{\rm{M}}} > {Q}_{S2}$). Under these circumstances, the concept of directional flow control differs only slightly from that of a current generator, where a current is applied to two resistors connected in parallel.

The devices presented in this work demonstrate a transition from no flow preference to flow preference along specific pressure-controlled pathways. Being able to control flow preference could enable microfluidic elements to actively change the preferred flow direction based on pressure control, through the intelligent interplay of the elastic elements and channel widths of different dendritic levels and within them.

## Conclusions

The integration of thin membranes as elastic walls in microfluidic channels using a single replica molding step is a viable, robust, and easily implementable design-based method for controlling the local elasticity in microfluidic devices. The control of the local elasticity in these devices results in a pressure-responsive increase in flow compliance. This pressure-responsive behavior has been successfully demonstrated in complex, physiologically relevant, single-to-multiple point dendritic delivery channel systems. The presence of membranes throughout the dendritic structure leads to a substantial, non-linear enhancement in flow when compared to structures lacking membranes.

While a maximum flow increase of ~60 % was achieved in this study, modifications to the geometric parameters of the membranes could enhance these results further. The maximum deformation of an elastic membrane is related to its thickness by ${w}_{\max }\propto {c}^{-3}$ and to its height by ${w}_{\max }\propto {b}^{4}$. Therefore, it is postulated that tuning the aspect ratio of the membranes $b/c$ can enhance the improvement factor. Fabricating the device with materials, which offer considerably higher achievable aspect ratios than PDMS, could lead to this enhancement. Additionally, since ${w}_{\max }\propto a$, the membrane length, the latter can also be tuned to increase the improvement factor. As the maximum deformation depends on Young’s modulus, ${w}_{\max }\propto {E}^{-1}$, controlling the cross-linking density or plasticizing silicone elastomers to achieve a significantly lower Young’s modulus, could be another way to increase the improvement factor by an order of magnitude^56–58^.

Moreover, the interaction of different working fluids with the membrane material (e.g., swelling via exposure to a good solvent like isopropanol for PDMS), can lead to the formation of stress-deformation states enhancing the improvement factor. A similar outcome could be achieved by manipulating temperature using a more thermally expansive material. Bonding the proposed design covalently with a thin elastic membrane as a top layer could also increase the improvement factor. The integration of these approaches, in a composite system, holds considerable promise for further enhancing the improvement factor. Such a system, could facilitate a pressure, solution, and temperature-responsive mechanism, thereby enhancing fluid flow through a microfluidic system. However, the fabrication process for such a composite system would be considerably more complex than that of replica molding.

Local elasticity control induces pressure-driven flow preferentiality along specific pathways in a flow-directing dendritic design. This method for controlling local elasticity in microfluidic systems reduces the pumping power requirements of such systems. It could be well-suited for platforms studying physiological phenomena, such as the interactions between viscoelastic fluids (e.g., blood) and elastic dendritic networks (e.g., the vasculature of animals and plants), where targeted flow distribution in elastic fluidic networks is of substantial significance^36,53^.

## Methods

### Preparation of microfluidic devices

A photomask design of all devices was drawn in AutoCAD 2019 (Autodesk, San Francisco, California, United States). The designs were then sent to Micro resist technology (Berlin, Germany), who fabricated the master mold using their SU-8 photoresist on silicon substrate microfabrication protocol, resulting in a 4′′ master mold. The photomask required for the master mold was a 5′′ chrome photomask (Compugraphics, Jena, Germany) for UV lithography. A 10:1 PDMS to curing agent mixture was prepared using the components of the SYLGARD 184 elastomer kit (Dow Silicones Deutschland, Wiesbaden, Germany). The mixture was stirred vigorously until it was white due to the incorporation of air. It was then degassed until no bubbles were present. The master mold was then placed in a Petri dish (diameter: 11 cm) and the PDMS mixture was poured over it until it was covered. The covered master was then degassed again to remove any air bubbles created during the casting step. The Petri dish was subsequently placed in an oven at approximately 65 ^o^C to allow the PDMS to cure overnight. The PDMS replica was then carefully removed from the master mold using a scalpel and tweezers. Individual replica devices were cut from the replica and connection holes were punched using a 1 mm diameter disposable biopsy punch (pfm medical, Köln, Germany). Throughout the process, care was taken to avoid damaging the structures of the replica devices. The individual replica devices were then placed in a container filled with a large volume of ethyl acetate (VWR International, Rosny-sous-Bois, France) to remove excess non-crosslinked PDMS oligomers. The replicas were left in ethyl acetate for 24 h, during which time the ethyl acetate was changed twice. At the end of the washing step, the ethyl acetate was removed and the replica devices were placed in a Petri dish with the structures side up. The replica devices in the Petri dish were then subjected to a further degassing step overnight to remove any residual ethyl acetate in the PDMS. The ethyl acetate exposure and subsequent reduced pressure drying of the devices serves an additional purpose. Most high aspect ratio structures on replica devices come off the master mold collapsed, but not destroyed. This is due to the elastic nature of PDMS and the lack of sufficient support in high aspect ratio PDMS structures, leading to stresses being developed in the structures during the curing and replica removal steps^59^. Ethyl acetate, which is a good PDMS solvent, swells the matrix and restores the geometry of the structures by removing these stresses. Gentle removal of the ethyl acetate by evaporation then allows the structures to retain their shape, even if they are not swollen. After complete evaporation of the ethyl acetate, the glass slides (R. Langenbrinck, Emmedingen, Germany) were placed in a bubble bag (BµB clean, Enschede, The Netherlands) containing isopropanol (Karl Roth, Karlsruhe, Germany) and then placed in an ultrasonic bath (BANDELIN electronic, Berlin, Germany) filled with water. The ultrasonic bath was subsequently switched on for 15 min to clean the surface of the glass slides. Note that a sufficient number of glass slides were placed in the bath to avoid mutual contact during the procedure. After the cleaning step, the slides were removed from the isopropanol and carefully dried on both sides by exposing them to a stream of nitrogen from a nitrogen gun. Next, the glass slides and replica devices (structure side up) were transferred to a plasma cleaner (Diener electronic, Ebhausen, Germany) and activated by exposure to ambient air plasma at a pressure ≤10 mbar. The side of the replica devices with the structures was covered with the activated glass slides. The partially bonded devices were placed on an IKAMAG RCT heating plate (IKA, Staufen, Germany) set at 200 ^o^C for 5 min to promote covalent bonding. At the end of this period, the bonding of all devices was checked under a BX61 optical microscope (Olympus, Tokyo, Japan) in reflection mode. The devices appeared to be well bonded and ready for flow experiments.

### Preparation of solutions

A solution of Pluronic F-127 (Sigma-Aldrich, Burlington, Massachusetts, USA) and distilled water (s1) at a concentration of 0.01 % w/w was prepared by vigorously mixing the two components until the Pluronic was completely dissolved in water. A portion of this solution was transferred to a 20 ml glass syringe (Trajan Scientific and Medicalm, Victoria, Australia). The working solution (s2) was prepared by vigorously mixing 50 ml of s1 in a vial with ≅ 0.045 g fluorescein sodium salt (Sigma-Aldrich, Burlington, Massachusetts, USA) and ≅ 0.1 g solution of polystyrene beads with a mean diameter of 1.1 µm (Sigma-Aldrich, Burlington, Massachusetts, USA). Solution s2 was then degassed. The degassed solution was then filtered and transferred to a 50 ml centrifuge tube (Heathrow Scientific, London, England) using an OB1 MK3+ pressure pump (Elveflow, Paris, France) and a 5 µm Millex syringe-driven filter unit (Merck, Darmstadt, Germany).

### Device pre-preparation and cleaning

Before the working fluid was transferred to a device, the device was first connected via PTFE tubing (Reichelt Chemietechnik, Heidelberg, Germany) to a 20 ml glass syringe containing s1, with a 0.2 µm Millex syringe-driven filter unit (Merck, Darmstadt, Germany) between the syringe and the device. The outlet of the device was subsequently connected to a waste container via PTFE tubing with an internal diameter of 0.4 mm (Reichelt Chemietechnik, Heidelberg, Germany). The syringe was then placed on a BASE 120 syringe pump (Cetoni, Korbussen, Germany) and flow rates of up to 80 µl/s were applied through the device. During this step, the walls of the device were coated with Pluronic F-127 to prevent unwanted particles from accumulating on them. The same procedure was repeated at the end of each experiment to remove as much fluorescein sodium salt residue and particles from the channels of the device as possible.

### Fluorescence measurements

Prior to performing any flow experiments, the pressure pump system was connected to the 50 ml centrifuge tube filled with solution s2, which served as the working fluid reservoir. The reservoir was connected to the device via two inlet ports (not visible in the following figures) and a single outlet port was connected to a Petri dish acting as a waste container. All connections were made using PTFE tubing with an internal diameter of 0.4 mm, silicone tubing with an internal diameter of 0.76 mm (microfluidic ChipShop, Jena, Germany) and an Elveflow luer kit (Elveflow, Paris, France). The outlet tubing was submerged in sufficient water to cover it. The device was then mounted on the transmission microscope Olympus BX61 and a 20x LMPlanFI objective (Olympus, Tokyo, Japan) was selected for imaging in combination with a U-TV0.63XC camera adapter. A SOLIS-3C LED (Thorlabs, Bergkirchen, Germany) was used as the transmission light source. A DMK 33UX174 CCD camera (The Imaging Source, Bremen, Germany), mounted on the microscope and connected to a PC with 16 GB RAM (Fujitsu, Kawasaki, Japan), with the camera frame rate set to 30 fps and the exposure time set to 1/10000 s or less, was used to focus on the center plane of the device and to track the polystyrene beads. This allowed the relative position of the working fluid reservoir and the fluid level in the waste container to be adjusted so that a minimal pressure difference was established between the inlet and outlet prior to each measurement. At this point, the beads were only moving due to Brownian motion.

The microscope was subsequently set to reflection mode, the transmission light source was turned off, the reflection light source was turned on, and a U-MBF3 reflected light brightfield mirror cube (Olympus, Tokyo, Japan) was inserted between the reflection path and the device. A SOLIS-1D LED (Thorlabs, Bergkirchen, Germany) was used as the reflection light source. A pressure profile was then applied to the device using the pressure pump. A video of the device under flow was recorded. The frame rate was set to either 10 or 30 fps, but the exposure time was set to 1/169 s so that the detected fluorescence signal was sufficiently strong.

After each measurement, the reflection light was switched off, the transmission light was switched on, the exposure time was again set to 1/10000 s or less, and the system was allowed to relax for a sufficient period of time to ensure that any elastic elements present in the system returned to their initial (non-buckled) state before the next measurement was taken. For each subsequent experiment, a pressure profile with a different maximum pressure was used and priority was given to establishing a minimum pressure difference between the inlet and outlet of the setup before taking measurements.

### PIV Measurements

PIV measurements were conducted in transmission microscopy mode on areas of the device with larger cross-sections, further down from three of the endpoints of the dendritic structure. A FASTCAM SA7 camera (Photron Deutschland, Reutlingen, Germany) was used to record approximately the last 2.3 s of steady flow for each different value of applied pressure. The video recording was conducted at frame rates of either 7500 or 10000 fps. Depending on the applied pressure $\Delta p$, some frames were skipped during the analysis to reduce the analysis time if the particle velocities were slow enough to allow for this. The exposure time was set to 1/100000 s. The objective used in the PIV experiments was a 10x LMPlanFI objective (Olympus, Tokyo, Japan).

## Data Availability

The data that support the findings of this study are available from the corresponding author upon reasonable request.
